# Lattice Matching (LM)—Prevention of Inadvertent Duplicate Publications of Crystal Structures

**DOI:** 10.6028/jres.107.036

**Published:** 2002-10-01

**Authors:** Alan D. Mighell

**Affiliations:** National Institute of Standards and Technology, Gaithersburg, MD 20899-8520

**Keywords:** crystallography, identification, lattice matching, mathematical lattices, multiple publication of the same structure, reduction

## Abstract

Lattice-matching techniques have proved to be extremely effective for the identification of unknown crystalline materials. A commonly employed lattice-matching strategy is based on matching the reduced cell of an *unknown* against a *database* of known materials represented by their respective standard reduced cells. The success of the method relies on the fact that the lattice or the lattice plus chemical information (e.g., element types) is highly characteristic of a material—like a fingerprint. Because of its intrinsic power, the procedure has many and diverse applications—in materials characterization, in nano-technology, in epitaxial growth, in materials design, etc. An especially fruitful role for the method is in the journal publication process as the quality of the scientific literature can be enhanced. The focus herein is on the major role that lattice matching can play in the prevention of inadvertent duplicate publications of the same structure and in the determination of key cross-references.

## 1. Introduction—Lattice Matching (LM)

**Fig. 1:**
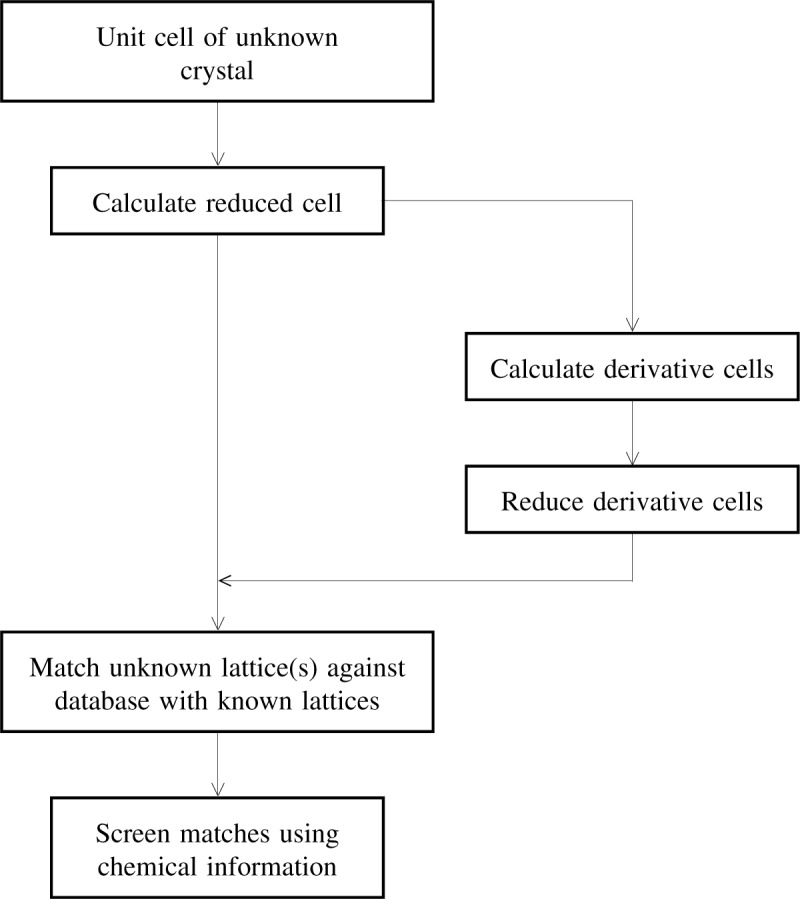
Identification via lattice matching (LM). A commonly employed version of lattice matching is based on matching the reduced cell of an *unknown* against a *database* of known materials represented by their respective standard reduced cells.

Lattice matching techniques have played a vital role in the identification of unknown crystalline materials. A commonly employed strategy for lattice matching [1–3]—based on matching the reduced cell of an *unknown* against a *database* of known materials represented by their respective standard reduced cells—is summarized in Fig. 1. As the figure shows, first lattice matching is carried out and then this is followed, if necessary, by a chemical screening of the resultant matches. (For convenience, this reduction based lattice-matching identification procedure is hereafter referred to as LM.) The success of LM relies on the fact that the lattice or the lattice and chemical information (e.g., element types) uniquely defines a crystalline material—like a fingerprint.

LM has proved to be a simple, powerful, and an easy-to-use method to identify unknowns. Practical experience has shown that the method is highly selective—even when the identification is against a database with several hundred thousand materials. Today the scientific community routinely uses LM in the identification of unknown crystalline compounds, as the strategy has been integrated into commercial x-ray diffractometers [4]. Similarly, LM—integrated into database distribution software—is routinely used in identifying unknowns against the various crystallographic databases.

Because of the intrinsic power of LM to identify and characterize materials, it has many diverse applications—e.g., in nano-technology, in epitaxial growth, in materials design, etc. An especially fruitful role for LM—the focus herein—is to enhance the journal publication process in crystallography and improve the quality of the scientific literature.

## 2. Discussion

During the experimental and publication process, it is critical to be aware of previous publications as well as contemporary work on the same or related materials. This knowledge is essential to enhance the expeditious use of previous research, thereby reducing unnecessary duplicate efforts, to optimize the information management of independent studies of the same material, and to provide key cross-references. However, inspection of the recent literature reveals that redundant efforts and inadvertent omission of key cross-references are not uncommon. The following three cases demonstrate the manner in which LM can prevent such problems in the first place or resolve them after publication.

### 2.1 Case 1. Piperidinium Dihydrogenphosphate

In 2001, a paper [5] reported the crystal structure of piperidinium dihydrogenphosphate as a “new compound.” LM (Fig. 1) reveals that the compound was previously reported in the literature in 1989 [6] (see Table 1). Both structures are the same even though the original structure was reported as monoclinic and the later structure reported, incorrectly, as triclinic. This example demonstrates that LM—applied during the course of the experimental work (or the publication process) for the 2001 paper—would have identified prior work and prevented an error in symmetry determination.

### 2.2 Case 2. N, N′-Diphenylguanidine

In 1999, the structure of orthorhombic N, N′-diphenylguanidine [8] was reported as a “new crystal structure.” LM reveals that this structure was previously reported in the literature in 1998 [9] (see Table 2). The later paper does not reference the earlier publication even though the structures are identical. In this case, however, most of the research for the later paper may have been carried out prior to the appearance in print of the 1998 paper. Cases like this are not uncommon because crystallographic data are published in such diverse fields and journals, and often it is especially difficult to find relevant recent publications. (Consequently, as a side issue, it would be useful to develop some mechanism so researchers can readily locate recent and concurrent work on a specified crystal structure.)

### 2.3 Case 3. 1,8-Terpin

It is not uncommon for a compound to be reported in a space group having too low symmetry. Fortunately, such errors are periodically located and corrected in the scientific literature. Accordingly, this was done [10] for an incorrect determination of 1,8-terpin [11]. This case, however, illustrates a trap because the focus was to correct the incorrect structure without realizing that the correct structure already had been published [12].

LM reveals that five papers report data on 1,8-terpin—two with lattice data only and three with full structure determinations. The lattice parameters for the three full structure determinations [10-12] are given in chronological order (left to right) in Table 3. Note that for Lattice II, the compound is described in a *C*-centered monoclinic space group. However, inspection of Table 3, reveals that the reduced form (reduced form number = 16) corresponds to an *F*-centered orthorhombic lattice. In the final study (Lattice III), Marsh and Herbstein correct the symmetry assigned to Lattice II and refine the structure in the *F*-centered orthorhombic lattice. It is instructive to note that the authors of the second [11] and third [10] determinations do not reference the first [12] determination, which was originally correct! This example demonstrates that routine application of LM—applied during the course of the experimental work (or the publication process) in the later studies [10,11]—would have prevented unintended multiple publications of the same structure, the error in symmetry determination, and the later effort that corrected the error in symmetry determination.

## 3. Conclusion—Enhancement of the Publication Process via LM

The above examples reveal that a significant problem in the publication process exists and that positive action needs to be taken to prevent unintended multiple publications of the same structure. Fortunately, LM comprises a simple and very powerful method to identify the same or related compounds. Clearly, the key advantage of *routine* application of LM is that the researcher is oriented with respect to previous work on the same and related structures. Consequently, as an integral part of the experimental and publication process, standard procedure dictates that LM should be routinely applied—especially at two key points of the process. First, by the experimentalist as soon as a unit cell has been determined and second, by the journal editor prior to acceptance of the manuscript for publication. For convenience, this identification procedure can be fully automated at both these points.

## Figures and Tables

**Table 1:** Crystallographic parameters reported for piperidinium dihydrogenphosphate (C_5_H_10_NH_2_·H_2_PO_4_) [5,6]. Comparison of the reduced cell parameters shows that the two structures are the same. Numbers in parentheses represent standard deviations

	Piperidinium dihydrogenphosphate	

	Lattice I	Lattice II

	Literature cells	
Cell	Cell 1	Cell 2
*a*(Å)	6.2397(6)	8.385(2)
*b*(Å)	8.4191(7)	6.227(2)
*c*(Å)	8.8523(2)	8.836(4)
*a*(°)	112.485(4)	90.0
*β*(°)	89.992(4)	112.43(3)
*γ*(°)	90.104(7)	90.0
*V*(Å^3^)	429.68(5)	426.4
System	Triclinic^a^	Monoclinic
Sp. Gr.	*P*1	*P*2_1_
Yr. Pub.	2001	1989
Ref. No.	[5]	[6]

	Reduced cells	

Cell	R-Cell 1	R-Cell 2
*a*(Å)	6.2397	6.227
*b*(Å)	8.4191	8.385
*c*(Å)	8.8523	8.836
*a*(°)	112.485	112.43
*β*(°)	89.992	90.0
*γ*(°)	90.104	90.0
*V*(Å^3^)	429.68	426.4

a Cell 1 of Lattice I was reported as triclinic. However, the reduced form No. 35 [7] for Cell 1 shows that the lattice is metrically monoclinic.

**Table 2:** Crystallographic parameters reported for orthorhombic N, N′-diphenylguanidine [8,9]. A comparison of the reduced cell parameters reveals that the two structures are the same. Numbers in parentheses represent standard deviations

	N,N′-diphenylguanidine	

	Lattice I	Lattice II

	Literature cells	
Cell	Cell 1	Cell 2
*a*(Å)	9.003(5)	12.653(5)
*b*(Å)	12.699(3)	20.54(2)
*c*(Å)	20.522(8)	8.944(5)
*α*(°)	90.0	90.0
*β*(°)	90.0	90.0
*γ*(°)	90.0	90.0
*V*(Å^3^)	2346.3(17)	2324(2)
System	Orthorhombic *P*	Orthorhombic *P*
Sp. Gr.	*P*2_1_2_1_2_1_	*P*2_1_2_1_2_1_
Yr. Pub.	1999	1998
Ref. No.	[8]	[9]

	Reduced cells	

Cell	R-Cell 1	R-Cell 2
*a*(Å)	9.003	8.944
*b*(Å)	12.699	12.653
*c*(Å)	20.522	20.540
*α*(°)	90.0	90.0
*β*(°)	90.0	90.0
*γ*(°)	90.0	90.0
*V*(Å^3^)	2346.3	2324.5

**Table 3:** Crystallographic parameters reported for 1,8-terpin (C_10_H_20_O_2_ H_2_O) in references [10–12]. Comparison of the reduced cell parameters reveals that all three compounds are the same. Numbers in parentheses represent standard deviations

	1,8-Terpin		

	Lattice I	Lattice II	Lattice III

	Literature cells		
Cell	Cell 1	Cell 2	Cell 3
*a*(Å)	10.930(2)	10.912(3)	18.421
*b*(Å)	18.425(5)	22.791(4)	22.791
*c*(Å)	22.791	10.705(2)	10.912
*α*(°)	90.0	90.0	90.0
*β*(°)	90.0	120.64	90.0
*γ*(°)	90.0	90.0	90.0
*V*(Å^3^)	4589.8	2290.6	4581.2
System	Orthorhombic *F*	Monoclinic *C*	Orthorhombic *F*
Sp. Gr.	*F*dd2	*C*c	*F*dd2
Yr. Pub.	1982	1986	1988
Ref. No.	[12]	[11]	[10]

	Reduced cells		

Cell	R-Cell 1	R-Cell 2	R-Cell 3
*a*(Å)	10.712	10.705	10.705
*b*(Å)	10.712	10.705	10.705
*c*(Å)	12.638	12.634	12.634
*α*(°)	102.74	102.71	102.71
*β*(°)	102.74	102.71	102.71
*γ*(°)	118.65	118.72	118.72
*V*(Å^3^)	1147.4	1145.3	1145.3

	Reduced forms		

Form	RF1	RF2	RF3
*A*·*a*	114.75	114.60	114.60
*b*·*b*	114.75	114.60	114.60
*c*·*c*	159.72	159.62	159.62
*b*·*c*	−29.87	−29.77	−29.77
*a*·*c*	−29.87	−29.77	−29.77
*a*·*b*	−55.00	−55.06	−55.06
Form No.	16	16	16

## References

[1] Mighell AD (1976). The Reduced Cell: Its Use in the Identification of Crystalline Materials. J Appl Cryst.

[2] Karen (Himes) VL, Mighell AD (1985). NBS*LATTICE: A Program to Analyze Lattice Relationships.

[3] Mighell AD, Karen VL (1986). Compound Identification and Characterization Using Lattice-Formula Matching Techniques. Acta Cryst.

[4] Bryam SK, Campana CF, Fait J, Sparks RA (1996). Using NIST Crystal Data within Siemens’ Software for Four-Circle and Smart CCD Diffractometers. J Res Natl Inst Stand Technol.

[5] Kaabi K, Ben Nasr C, Rzaigui M (2001). Synthesis and Crystal Structure of C_5_H_12_NPO_4_H_2_. J Solid State Chem.

[6] Aakeröy CB, Hitchcock PB, Moyle BD, Seddon KR, Novel A (1989). Class of Salts for Second Harmonic Generation. J Chem Soc, Chem Commun.

[7] Mighell AD (2001). Lattice Symmetry and Identification—The Fundamental Role of Reduced Cells in Materials Characterization. J Res Natl Inst Stand Technol.

[8] Paixão JA, Matos Beja A, Pereira Silva PS, Ramos Silva M, Alte Da Veiga L (1999). A new orthorhombic phase of N,N′- diphenylguanidine. Acta Cryst.

[9] Tanatani A, Yamaguchi K, Azumaya I, Fukutomi R, Shudo K, Kagechika H (1998). N-Methylated Diphenylguanidines: Conformations, Propeller-Type Molecular Chirality, and Construction of Water-Soluble Oligomers with Multilayered Aromatic Structures. J Am Chem Soc.

[10] Marsh RE, Herbstein FH (1988). More Space-Group Changes. Acta Cryst.

[11] Ho T-I, Cheng M-C, Peng S-M, Chen F-C, Tsau C-C (1986). Structure of Terpin. Acta Cryst.

[12] Suga T, Hirata T, Aoki T (1982). An X-ray Crystallographic Study on cis-trans Configurational Assignment to “cis-” and “trans-1, 8-terpins” and a Proposal of New Designation for Discriminating between the Configurational Isomers. Bull Chem Soc Jpn.

